# Oncological Outcomes of Adjuvant Radiotherapy for Partial Ureterectomy in Distal Ureteral Urothelial Carcinoma Patients

**DOI:** 10.3389/fonc.2021.699210

**Published:** 2021-09-30

**Authors:** Hong-zhen Li, Xiaoying Li, Xian-shu Gao, Xin Qi, Ming-Wei Ma, Shangbin Qin

**Affiliations:** Radiation Oncology, Peking University First Hospital, Beijing, China

**Keywords:** urothelial carcinoma, partial ureterectomy, radical nephroureterectomy, adjuvant radiotherapy, recurrence-free survival (RFS)

## Abstract

**Purpose:**

We retrospectively analyzed the oncological outcomes of T3 or G3 distal ureteral urothelial carcinoma (DUUC) underwent partial ureterectomy (PU) followed by adjuvant radiotherapy (ART).

**Methods:**

From January 2008 to September 2019, clinical data from a total of 221 patients with pathologic T3 or G3 who underwent PU or RNU at our hospital were analyzed. 17 patients of them were treated with PU+ART, 72 with PU alone and 132 with radical nephroureterectomy (RNU). Clinicopathologic outcomes were evaluated. Survival was assessed using the Kaplan-Meier method. Cox regression addressed recurrence-free survival (RFS), metastasis-free survival (MFS), cancer specific survival (CSS) and overall survival (OS).

**Results:**

Median age and follow-up time were 68 (IQR 62-76) years old and 43 (IQR 28-67) months, respectively. In univariate and multivariable analyses, no lymph node metastasis(LNM) and ART were independent prognostic factors of RFS (p=0.031 and 0.016, respectively). ART significantly improved 5-year RFS compared with the PU alone, (67.6% *vs*. 39.5%, HR: 2.431, 95%CI 1.210-4.883, p=0.039). There was no statistical difference in 5-year RFS between PU+ART and RNU groups (67.6% *vs*. 64.4%, HR=1.113, 95%CI 0.457-2.712, p=0.821). Compared with PU alone or RNU, PU+ART demonstrated no statistical difference in 5-year MFS (PU+ART 73.2%, PU 57.2%, RNU69.4%), CSS (70.7%, 55.1%, 76.6%, respectively), and OS (70.7%, 54.1%, 69.2%, respectively).

**Conclusions:**

For distal ureteral urothelial carcinoma patients with T3 or G3, adjuvant radiotherapy could significantly improve recurrence-free survival compared with partial ureterectomy alone. There was no significant difference between survival outcomes of PU+ART and radical nephroureterectomy.

## Introduction

The “gold-standard” treatment for urothelial carcinoma (UC) of the distal ureter (DUUC) is radical nephroureterectomy (RNU) with bladder cuff removal. But partial ureterectomy (PU) is also a viable option in selected cases, especially in cases with a solitary kidney or bilateral ureteral carcinoma or chronic renal insufficiency (1). The results of the meta-analysis showed that there was no significant difference in cancer specific survival (CSS) between patients with Ta/T1 and G1-G2 tumors after PU and RNU (2). For patients with pathologic T3 or G3, PU compared with RNU had worse recurrence-free survival (RFS), bladder recurrence, and overall survival (OS) (3, 4). The value of adjuvant radiotherapy (ART) for UC after RNU is still controversial (1). However, several studies from Asian countries showed that adjuvant radiotherapy after RNU can improve LRFS, distant metastasis free survival (DMFS) or even OS in patients with high-risk pathological factors (T3, G3) UC (5–8). It is worthwhile to investigate whether postoperative radiotherapy can improve the survival of patients with adverse factors such as G3 or T3 after PU.

In the present study, we retrospectively investigated the survival outcomes of patients with T3 or G3 DUUC treated with PU+ART versus PU alone or RNU.

## Materials and Methods

We identified all DUUC patients with pathological T3 or G3 who had undergone surgery (PU or RNU) from January 2008 to September 2019 at our hospital. Sample collection was approved by the Ethics Committee of Peking University First Hospital.

Inclusion criteria of the study were as follows: (1) pathologic diagnosis of urothelial carcinoma of distal ureter, (2) pathological stage T3N0-2 M0,or (3) pathological tumor grade G3, and (4) regular follow-up at our center. The exclusion criteria were as follows: (1) previous or sequential second primary cancers (except for UC), (2) evidence of metastatic disease at the time of diagnosis, and (3) patients with adjuvant chemotherapy.

The following data were reviewed: age at diagnosis, gender, type of surgery, tumor characteristics (AJCC TNM stage 8^th^ edition), tumor pathological grading (WHO 2004), surgical margin status, tumor site, maximum tumor diameter, disease recurrence, and metastatic progression.

The distal ureter was defined below the lower margin of the sacrum according to the standard definition in Campbell-Walsh Urology (9th Edition) (9).

In all, 246 consecutive patients were included. 3 patients with evidence of metastatic disease at the time of diagnosis were excluded. 22 patients lost to follow-up were excluded. Finally, a total of 221 patients were eligible for retrospective analysis.

According to the treatment modes, there were 17 cases in the PU+ART group, 72 cases in the PU alone group and 132 cases in the RNU group. In the PU+ART and PU alone groups, PU was indicated in imperative cases, such as patients with bilateral tumor (2 cases in PU alone group), chronic renal insufficiency (13 cases in PU+ART, 48 cases in PU alone), a solitary kidney (3 cases in PU+ART, 20 cases in PU alone), or prohibitive operative risk (American Society of Anesthesiologists score≥3) (1 in PU+ART, 2 in PU alone). In the PU+ART group, adjuvant radiotherapy was performed within 6 weeks after surgery using volumetric modulated arc radiotherapy (VMAT) with 6-MV photon. Clinical target volume (CTV) included at least 2cm above and below the anastomotic site after partial ureterectomy and 1cm around the ureter bed, including lymph node drainage area on the same layer. The radiotherapy prescription was 50Gy/25fractions/5weeks. Clinical target volume of tumor bed (CTVtb) was given simultaneously boost to 62.5Gy/25fractions/5weeks. The range and dose of radiation were consistent in all cases.

All the patients had regular follow-up every 3months for the first 2 years after surgery, every 6 months in the next 3 years, and annually thereafter. Evaluations included a history, physical examination, blood test, urinary cytology, chest radiograph, cystoscopy, and ultrasonograms or CT/magnetic resonance imaging (MRI) scans of the abdomen.

Disease recurrence was defined as any documented relapse in the regional lymph nodes, bladder, or urothelial tract. Metastatic progression was defined as any recurrence in the nonregional lymph nodes or the lung, liver, bone, and other organs. For the above new lesions or enlarged lymph nodes, cystoscopy, CT, or ultrasound-guided puncture biopsy was recommended according to the location of the lesions, to confirm the diagnosis. If the patient who was unwilling to or not suitable for biopsy, two different imaging examinations were recommended. If the new lesion was indicated by two or more imaging experts in different examinations, the clinical diagnosis of recurrence or metastasis can be made.

Clinical and pathological characteristics of the three groups were compared using either the Mann-Whitney U test or the chi-square test for continuous or categorical variables, respectively. Univariate and multivariate Cox proportional hazards regression analyses were performed to determine the association between risk factors and recurrence-free survival (RFS), metastasis-free survival (MFS), cancer-specific survival (CSS) and overall survival (OS). Variables identified as significant by the univariate analysis were considered for the multivariate analysis. We used the Kaplan-Meier to estimate RFS, MFS, CSS and OS, and the log-rank test was applied to compare survival curves between PU+ART and PU alone or RNU groups. We used the Statistical Package for Social Sciences (SPSS, version 22.0) and GraphPad Prism 8 to analyze all data. All tests were two-sided, and *p*<0.05 were considered to indicate statistical significance.

## Result

Median age of these patients were 68 (IQR 62-76) years old and the median follow-up time were 43 (IQR 28-67) months. Clinical and pathological characteristics of the three cohorts separately listed for variables are shown in **Table 1**. There was no statistically significant difference between PU+ART group and PU alone or NRU groups for the variables used for matching, including age, gender, tumor site, maximal tumor diameter, tumor grade, pT and pN stage. Positive surgical margin rates were higher in PU+ART groups, compared with PU alone or RNU groups (*p*= 0.026 and *p*= 0.012, respectively). There was significantly more lymph node metastasis in PU+ART groups, compared with PU alone (*p*= 0.034).

**Table 1:** Patient clinical and pathological characteristic.

Characteristics	PU Group (%)	*p* value (PU+ART vs PU)	PU+ART Group (%)	*p* value (PU+ART vs RNU)	RNU Group (%)
Patient number	72		17		132
Median age (IQR)	68 (62-75)	0.620	71 (63-78)	0.657	69 (62-76)
Gender		0.937		0.753	
Male	34 (47.3)		8 (47.1)		59 (44.7)
Female	38 (52.7)		9 (52.9)		73 (55.3)
Site		0.892		0.933	
Left	39 (51.3)		9 (52.9)		68 (51.5)
Right	31 (43.1)		8 (47.1)		64 (48.5)
Bilateral	2 (2.8)		0 (0)		0 (0)
Maximal tumor diameter (Range)	1.9 (0.2-4.8)	0.635	2.5 (0.6-4.5)	0.787	2.5 (0.5-15.0)
Tumor grade		0.064		0.189	
G2	10 (13.9)		5 (29.4)		26 (19.7)
G3	62 (86.1)		12 (70.6)		106 (80.3)
Pathological stage		0.732		0.353	
T1	7 (9.7)		2 (11.8)		7 (5.3)
T2	23 (31.9)		5 (29.4)		37 (28.0)
T3	42 (58.3)		10 (58.8)		83 (62.8)
T4	0 (0)		0 (0)		5 (3.8)
Lymph node Meta.		**0.034**		0.121	
pNx	58 (80.5)		14 (82.4)		99 (75.0)
pN0	11 (15.3)		1 (5.9)		15 (11.4)
pN1	3 (4.2)		2 (11.8)		7 (5.3)
pN2	0 (0)		0 (0)		11 (8.3)
Surgical Margin		**0.026**		**0.012**	
Positive	5 (6.9)		4 (28.6)		6 (4.5)
Negative	67 (93.1)		13 (71.4)		126 (95.5)
Median follow-up (months) (IQR)		0.680		0.785	
	44 (28-57)		47 (29-76)		43 (31-67)

Bold words represent p value < 0.05, which is statistically significant.

**Figure 1:**
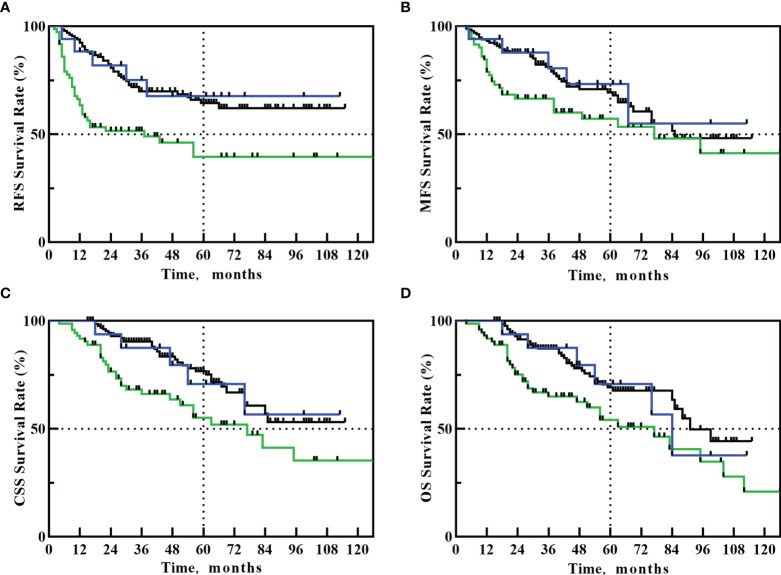
Kaplan–Meier curves for: RFS **(A)**, MFS **(B)**, CSS **(C)**, OS **(D)** after PU+RT (blue line), PU alone (green line) or RNU (black line).

The 5-year RFS, MFS, CSS, and OS in PU+ART group were 67.6%, 73.2%, 70.7% and 70.7%, respectively. The 5-year RFS, MFS, CSS, and OS in PU alone group were 39.5%, 57.2%, 55.1% and 54.1%, respectively. The 5-year RFS, MFS, CSS, and OS in RNU group were 64.4%, 69.4%, 76.6% and 69.2%, respectively. Compared with the PU alone group, PU+ART significantly improved 5-year RFS (67.6% *vs*. 39.5%, HR: 2.431, 95%CI 1.210-4.883, *p*=0.039). However, there was no statistical difference in 5-year RFS between the PU+ART and RNU groups (67.6% *vs*. 64.4%, HR=1.113, 95%CI 0.457-2.712, *p*=0.821). Compared with PU alone group, PU+ART group demonstrated no statistical difference in 5-year MFS (73.2% *vs*. 57.2%, *p*=0.144), CSS (70.7% *vs*. 55.1%, *p*=0.131) and OS (70.7% *vs*. 54.1%, *p*=0.121). Compared with RNU group, PU+ART group exhibited similar 5-year MFS (73.2% *vs*. 69.4%, *p*=0.778), CSS (70.7% *vs*. 76.6%, *p*=0.907) and OS (70.7% *vs*. 69.2%, *p*=0.985). RFS, MFS, CSS and OS Survival Kaplan-Meier curves are drawn in **Figures 1A–D**.

During the follow-up, 5 of the 17 patients with PU+ART had recurrence, all the recurrence occurred in the bladder, and the median recurrence time was 17 months (5-38 months). The median RFS time of the PU+ART group had not yet been reached. Of the 72 patients with PU alone, 38 patients had recurrence, with a median recurrence time of 9 months (2-56 months), and a median RFS time of 37 months in this group. Of the 132 patients with RNU, 41 patients had recurrence, with a median recurrence time of 23 months (5-66 months), and a median RFS time had not yet been reached in this group.

5 of the 17 patients with PU+ART had metastases, and the median metastasis time was 36 months (5-67 months) in the 5 cases. The median MFS time of the PU+ART group had not yet been reached. Of the 72 patients with PU alone, 30 patients had metastases, with a median metastasis time of 19 months (4-95 months), and a median MFS time of 77 months in this group. Of the 132 patients with RNU, 42 patients had metastases, with a median metastasis time of 31 months (5-85 months), and a median MFS time of 85 months in this group.

Because the difference in survival outcomes only existed in the RFS between PU+ART and PU groups. To find patients who may benefit from adjuvant radiotherapy after PU. In 89 patients underwent PU, univariate analyses were performed for local recurrence by age (≤70 years *vs*. > 70 ypears), gender, left and right sides, tumor diameter, G grade, pT stage, pN stage (lymph node metastasis or not), positive surgical margin, adjuvant radiotherapy. These analysis results were shown in **Table 2**. On univariable analyses, Tumor Diameter>2.5cm, pN+, G3, positive surgical margin, and non- ART were all significantly associated with increased local recurrence for patients with PU (p< 0.05). On multivariable analyses, only pN+ (HR= 1.625; 95% CI 1.063-2.372, p=0.031), Non- ART (HR =2.590; 95% CI 1.855-3.636, P = 0.016) remained independent predictors of increased local recurrence.

**Table 2:** Univariable and multivariable Cox regression analyses predicting local recurrence in 89 patients.

Variable	Univariate analyses			Multivariate analyses		
	HR	95%CI	*p* value	HR	95%CI	*p* value
Age						
>70 years	1.645	0.653-4.142	0.618			
≤70 years	1					
Gender						
Male	1.223	0.314-2.126	0.675			
Female	1					
Site						
Left	1.145	0.386-1.918	0.937			
Right	1					
Tumor diameter						
>2.5cm	2.953	1.146-4.738	**0.008**	1.422	0.866-2.342	0.164
≤2.5cm	1					
pT stage						
T3-4	1.580	0.894-4.202	0.559			
T2	1					
G分级						
G3	1.073	0.155-7.462	0.943			
G2						
pN stage						
N+	2.659	1.631-5.172	**0.016**	1.625	1.063-2.372	**0.031**
N0						
Surgical Margin						
Positive	2.816	1.804-4.646	**0.012**	1.203	0.884-2.132	0.563
Negative	1					
ART						
Yes	1		** **	1		
No	2.433	1.209-4.878	**0.039**	2.590	1.855-3.636	0.016

Bold words represent p value < 0.05, which is statistically significant.

The toxicity of patients with adjuvant radiotherapy was mild, no severe toxicity was observed during radiotherapy. After 3-6 months of radiotherapy, more than 80% of the patients’ toxicity disappeared completely. In long-time follow-up, a few patients had late adverse reactions, such as frequent urination, diarrhea, leucopenia, thrombocytopenia, etc., which did not affect the quality of life. Detailed toxicity is listed in **Table 3**.

**Table 3:** Acute and late toxicity in 17 patients of adjuvant RT (NCI-CTCAE V4.0).

Toxicity/ No. (%)		Grade 0	Grade 1	Grade 2	≥Grade 3
Acute	Urination urgency-frequency	4 (23.5)	8 (47.1)	5 (29.4)	0 (0)
	Nausea and vomiting	7 (41.2)	7 (41.2)	3 (17.6)	0 (0)
	Abdominal pain and diarrhea	5 (29.4)	10 (58.8)	2 (11.8)	0 (0)
	Cytopenia	6 (35.3)	6 (35.3)	5 (29.4)	0 (0)
Late	Urination urgency-frequency	14 (82.4)	3 (17.6)	0 (0)	0 (0)
	Chronic diarrhea	15 (88.2)	2 (11.8)	0 (0)	0 (0)
	Cytopenia	15 (88.2)	2 (11.8)	0 (0)	0 (0)

## Discussion

To our understanding, this is the first retrospective study on the adjuvant radiotherapy cohort after partial ureterectomy for distal ureteral urothelial carcinoma. UTUCs are relatively rare, that account for less than 5% of all genitourinary diseases in USA (10). Therefore, the quantity of clinical studies related to UTUC is limited. There have been fewer studies on UTUC postoperative radiotherapy.

Partial ureterectomy (PU) has been developed in patients with imperative indications, such as patients with a solitary kidney, chronic renal insufficiency, bilateral tumor, or prohibitive operative risk. Due to surgical techniques, cases of distal ureteral urothelial carcinoma (DUUC) are more suitable for PU. PU can improve postoperative renal function compared with RNU (11), but the key to cancer treatment is to prolong survival. In high-risk cases, PU is likely to have a worse survival than RNU (3, 4). The aim of postoperative radiotherapy is to reduce the risk of local recurrence after surgery, thereby inhibiting distal metastasis and improving survival. It is worthwhile to investigate whether adjuvant radiotherapy can improve the survival of patients with PU.

We focused on cases with poor biological behavior, such as T3 or G3. In cases of UTUC with low stage or grade, the survival outcomes obtained by PU and RNU were similar (12), so the value of radiotherapy is not significant. On the contrary, in T3 or G3 patients, PU generally had worse survival. In one study (3), RNU demonstrated superior progression-free survival (PFS) for G3 disease (88.9% *vs* 55.6%, p= 0.033). In another large multicenter study (12), pT stage demonstrated to be an independent prognostic factor of RFS and MFS in patients after PU. Compared with non-muscular infiltrating tumors, pT3 increased the risk of local recurrence and distal metastasis up to 163% (95%CI: 1.08- 2.46, p=0.01) and 430% (95%CI:1.97- 9.35, p<0.001), respectively. However, several studies showed that adjuvant radiotherapy can improve RFS, DMF or even OS in patients with high-risk pathological factors (T3, G3) UTUC (5–8). Jwa (5) found ART could improve RFS, and Chen (6) found a benefit from ART in OS. In previous study (8), we found that patients with local recurrence had poorer CSS (4-year CSS rate 36 ± 7.5% *vs* 88.4 ± 2.2%, *p* =0.000), and adjuvant radiotherapy could reduce the local recurrence (HR = 0.177; 95% CI 0.064-0.493, *p*=0.001).

The potential decrease in survival caused by PU compared with RNU can be compensated by adjuvant radiotherapy. In the present study, PU+ART significantly improved 5-year RFS (67.6% *vs*. 39.5%, HR: 2.431, 95%CI 1.210-4.883, *p*=0.039) compared with the PU alone group. Although there was no statistical difference in MFS, CSS, and OS between the PU+ART group and the PU group, the survival benefit trends were seen numerically. The statistical difference was limited by the small number of cases in the PU+ART group. At the same time, the results show that the PU+ART group is very similar to RNU in terms of RFS, MFS, CSS, and OS.

Another interesting finding of the present study was the prognostic impact of pathological lymph node status. LNM was independent prognostic factors of RFS (p=0.031) in multivariate analysis. In previous study (8), we found that LNM was independent predictors of RFS. Colin et al (12) showed that LNM was independent prognostic factors of CSS. some authors have described the prognostic value of G3 or T3 for OS and CSS in multivariate analysis. In this study, we strictly defined all enrolled patients as T3 or G3.

The value of adjuvant chemotherapy after UTUC was controversial in our hospital, so few cases are treated with adjuvant chemotherapy. A small number of patients who had received adjuvant chemotherapy were excluded from this retrospective study, because the impact of adjuvant chemotherapy on survival outcome analysis was avoided.

However, there were still some limitations to our study. First, this was a retrospective study. Second, the number of cases in PU+ART groups were limited. Third, in this study, local recurrence or metastases were based on medical images, and there was no pathological verification of lesions. In the future, the sample size of the cohort can be expanded.

## Conclusions

For distal ureteral urothelial carcinoma patients with T3 or G3, adjuvant radiotherapy could significantly improve recurrence-free survival compared with partial ureterectomy alone. There was no significant difference between survival outcomes of PU+ART and radical nephroureterectomy. A prospective database that would assess this question is urgently needed to reach a conclusion for this on-going debate.

## Data Availability Statement

The raw data supporting the conclusions of this article will be made available by the authors, without undue reservation.

## Ethics Statement

The studies involving human participants were reviewed and approved by Ethics Committee of Peking University First Hospital. The patients/participants provided their written informed consent to participate in this study.

## Author Contributions

(I) Conception and design: H-zL. (II) Administrative support: X-sG. (III) Provision of study materials or patients: H-zL, XL, M-wM. (IV) Collection and assembly of data: H-zL, XL, M-wM, SQ. (V) Data analysis and interpretation: H-zL, XL. All authors contributed to the article and approved the submitted version.

## Conflict of Interest

The authors declare that the research was conducted in the absence of any commercial or financial relationships that could be construed as a potential conflict of interest.

## Publisher’s Note

All claims expressed in this article are solely those of the authors and do not necessarily represent those of their affiliated organizations, or those of the publisher, the editors and the reviewers. Any product that may be evaluated in this article, or claim that may be made by its manufacturer, is not guaranteed or endorsed by the publisher.
